# Combination immunotherapy: a road map

**DOI:** 10.1186/s40425-017-0218-5

**Published:** 2017-02-21

**Authors:** Patrick A. Ott, F. Stephen Hodi, Howard L. Kaufman, Jon M. Wigginton, Jedd D. Wolchok

**Affiliations:** 1 Dana-Farber Cancer Institute, Harvard Medical School, 450 Brookline Avenue, Dana540C, Boston, MA 02215 USA; 2 Rutgers Cancer Institute of New Jersey, 195 Little Albany Street, New Brunswick, NJ 08901 USA; 3 MacroGenics, Inc., 9640 Medical Center Drive, Rockville, MD 20850 USA; 4 Memorial Sloan Kettering Cancer Center, 1275 York Avenue, Z-1503, New York, NY 10065 USA

**Keywords:** Immunotherapy, Combination, Checkpoint inhibitors, Preclinical models, Clinical trial, Endpoints

## Abstract

Cancer immunotherapy and in particular monoclonal antibodies blocking the inhibitory programed cell death 1 pathway (PD-1/PD-L1) have made a significant impact on the treatment of cancer patients in recent years. However, despite the remarkable clinical efficacy of these agents in a number of malignancies, it has become clear that they are not sufficiently active for many patients. Initial evidence, for example with combined inhibition of PD-1 and CTLA-4 in melanoma and non-small cell lung cancer (NSCLC), has highlighted the potential to further enhance the clinical benefits of monotherapies by combining agents with synergistic mechanisms of action. In order to address the current progress and consider challenges associated with these novel approaches, the Society for Immunotherapy of Cancer (SITC) convened a Combination Immunotherapy Task Force. This Task Force was charged with identifying and prioritizing the most promising prospects for combinatorial approaches as well as addressing the challenges associated with developing these strategies. As a result of the extensive clinical benefit and tolerable side effects demonstrated with agents inhibiting the PD-1 pathway, an overview of current evidence to support its promising potential for use as a backbone in combination strategies is presented. In addition, key issues in the development of these strategies including preclinical modeling, patient safety and toxicity considerations, clinical trial design, and endpoints are also discussed. Overall, the goal of this manuscript is to provide a summary of the current status and potential challenges associated with the development and clinical implementation of these strategies.

## Background

The strategy of using monoclonal antibodies against inhibitory receptors on immune cells, termed immune-checkpoint blockade, for the treatment of cancer has triggered substantial enthusiasm among clinicians, scientists, and patients [1]. The efficacy of this approach was first proven in patients with advanced melanoma based on the improved overall survival (OS) of patients treated with the anti-cytotoxic T lymphocyte associated protein 4 (CTLA-4) directed monoclonal antibody ipilimumab [2, 3]. The impressive anti-tumor activity of PD-1/PD-L1 blockade not only in melanoma and renal cell cancer, but also in tumors previously not considered immune-responsive, particularly NSCLC reported initially in 2012, provided proof of concept for the efficacy of immunotherapy as a more broadly applicable tool for the treatment of cancer [4, 5]. Since then, PD-1/PD-L1 inhibition has demonstrated remarkable anti-tumor activity, including durable responses for several years, in a broad spectrum of solid and hematological malignancies, leading to regulatory approval of an increasing list of agents in a growing number of cancers. Nevertheless, the clinical efficacy of PD-1 pathway inhibition as monotherapy has been limited to subsets of patients in most tumor types studied to date, with response rates of 20% or less in many cancers including common types such as breast, colon, and prostate cancer. While predictive biomarkers such as PD-L1 expression on tumor and immune cells [6], mutational/neoantigen load [7–9], and inflammatory gene signatures [10, 11] may allow enrichment of patient populations that are responsive to this therapy, combination therapies will likely be required to enhance and broaden the anti-tumor activity of immune checkpoint inhibition.

## Agent prioritization

### Backbone of combination therapies

The established anti-tumor activity of PD-1/PD-L1 inhibition as monotherapy in a wide spectrum of cancers coupled with its favorable toxicity profile provides a strong rationale for its use as a backbone for combinatorial strategies. Despite the vastly accelerated pace of preclinical and clinical investigation of other cancer immunotherapy agents in recent years, this combination of broad single agent activity and tolerability seen with PD-1 pathway inhibition is so far unparalleled; there are no other compounds on the horizon that could take the place of PD-1 pathway inhibition for this purpose.

### Partnering agents with anti-PD-1/PD-L1 backbone

#### Guiding principles

There is emerging evidence that immune checkpoint blockade is effective primarily in tumors that are already recognized by the immune system, as manifest by a pre-existent CD8+ T cell infiltrate. Broadly speaking, the lack of a spontaneous tumor directed immune response may be because of the “invisibility” of the tumor to the immune system due to tumor antigens that are not sufficiently distinct from self-antigens; alternatively, tumor cell intrinsic oncogenic pathways may actively subvert an antitumor immune response as was shown for the β-catenin pathway [12]. Approaches that have the potential to convert a “non-T cell inflamed” tumor into a T cell inflamed tumor such as novel vaccines, oncolytic virus approaches, stimulation of co-stimulatory molecules, targeted therapy (such as BRAF/MEK inhibition in BRAF mutant melanoma, ALK inhibition in ALK–rearranged NSCLC), radiation/chemotherapy, and adoptive cell therapy (T cells, CARs) should be prioritized – particularly for tumor types that have shown little response to single agent anti-PD-1/PD-L therapy and for individual patients, ideally biomarker-selected, who have lower predicted response to the PD-1/PD-L1 backbone. Strategies that primarily address additional immunosuppressive mechanisms in the tumor microenvironment, such as indoleamine 2,3-dioxgenase (IDO)-inhibition, TGF-β blockade, regulatory T cell (Treg) depletion, and angiogenesis inhibition may be particularly effective to enhance or rescue tumor responses achieved with anti-PD-1/PD-L1 monotherapy.

#### Vaccines

Anti-PD-1 monotherapy may be limited by the size of the pool and specificity of pre-existent tumor directed T cells generated by physiological interaction of the evolving tumor and the host immune system. Particularly for tumors with low mutational burden, it is conceivable that insufficient numbers of T cell clones are spontaneously primed by tumor antigens, and therefore, a critical threshold of T cells is not reached to trigger an immune infiltrate. An effective vaccine may provide the necessary stimulation to broaden the repertoire of T cells engaged in the anti-tumor response. The accumulating evidence for tumor neoantigens as critical target antigens for tumor rejection coupled with the striking correlation of anti-CTLA-4 and anti-PD-1 induced tumor responses with the mutational/neoantigen load in NSCLC, melanoma, and microsatellite instability (MSI) high tumors make a strong case for vaccination targeting neoantigens [7–9, 13, 14]. The most effective vaccination strategies will aim to co-administer neoantigens, or other potential antigens that can be targets for T cell recognition (e.g., tissue differentiation or cancer stem cell associated antigens), with strong immune-adjuvants such as TLR agonists, possibly taking advantage of new delivery systems such as novel material engineered scaffolds.

#### Oncolytic viruses

The oncolytic virus talimogene laherparepvec (T-VEC) has shown systemic anti-tumor activity in advanced melanoma, leading to its regulatory approval in the United States, European Union, and Australia for the treatment of melanoma [15]. Similar to vaccination, oncolytic virus therapy has the potential to induce priming of T cells, leading to T cell mediated cytolysis of directly injected as well as distant tumor metastases. In phase I trials, the combination of T-VEC with ipilimumab as well as with pembrolizumab has shown higher objective tumor responses compared to ipilimumab and pembrolizumab monotherapy [16, 17]. Larger trials testing both combinations are ongoing.

#### Agonistic co-stimulatory antibodies

Costimulatory molecules such as CD137 (4-1BB), CD134 (OX40), glucocorticoid-induced TNFR (GITR; CD357), and CD40 are expressed by activated T cells, activated natural killer (NK) cells, natural killer T (NKT) cells, Tregs, and other immune cells [18]. Stimulation of these molecules can lead to increased effector functions (cytokine production and cytolytic activity), restrained suppressive activity of Tregs, enhanced cytokine secretion by NK cells, and enhanced antibody-dependent cellular cytotoxicity. Preclinical single agent anti-tumor activity including durable complete responses has been shown in murine tumor models as well as in early clinical trials [19, 20]. Combined activation of CD137 and inhibition of PD-1 was synergistic in an ovarian cancer model and is currently undergoing clinical testing [21, 22]. The inhibition of the immunologic checkpoint PD-1 and stimulation of costimulatory molecules are complementary strategies to enhance immune responses and therefore provide a strong rationale for use in combination.

#### Adoptive T cell therapy, CAR-T cell therapy

Adoptively transferred T cells generated from tumor TILs, T cells bearing engineered, tumor specific T cell receptors, and chimeric antigen receptor (CAR) T cells all have shown remarkable anti-tumor activity in select solid and hematological malignancies [23–25]. CAR T cells and T cells with engineered tumor specific TCRs may have the ability to induce an inflamed tumor microenvironment and therefore to be promising partnering strategies with PD-1/PD-L1 blockade.

#### CTLA-4 blockade/other checkpoint inhibitors

The combination of PD-1/PD-L1 inhibition with blockade of the non-redundant and complementary checkpoint CTLA-4 is backed by strong pre-clinical evidence and has proven very effective in advanced melanoma patients in phase I-III trials, as manifest by rapid, durable responses in a high proportion of patients [26–28]. This remarkable success provides the rationale for ongoing clinical investigation of combined PD-1/PD-L1 and CTLA-4 inhibition in many different tumor types and the exploration of PD-1/PD-L1 inhibition in combination with inhibition of other immune checkpoints such as Tim-3 and Lag-3.

#### Targeted therapy

Oncogenic pathway inhibition such as BRAF and MEK inhibition in melanoma has shown many effects on tumor and immune cells, including increased expression of melanoma differentiation antigens and HLA on melanoma cells, paradoxical activation of the MAPK pathway in T lymphocytes, induction of PD-L1 expression, and inhibition of suppressive cytokines such as IL-10 and VEGF [29]. Melanoma antigen expression in human tumor samples was decreased at the time of tumor progression and restored with subsequent combined MEK/BRAF inhibition [30]. Furthermore, increased CD8+ T cell tumor infiltration was observed in early tumor samples in patients treated with BRAF inhibition. This preclinical evidence supports the investigation of PD-1/PD-L1 inhibition with BRAF/MEK inhibition and potentially with other oncogenic pathway inhibitors. The restoration of other abnormal oncogenic signals in cancer cells, such as in the Wnt-β-catenin, AKT-PI3K-mTOR, and epidermal growth factor (EGF)/EGF receptor (EGFR) signaling pathways, may also be promising strategies for combination immunotherapy approaches.

#### Angiogenesis-inhibition

By facilitating both the growth of cancer cells and immune suppression, tumor angiogenesis is an important link between a tumor and the immune response directed against that tumor. VEGF modulates anti-tumor immunity on multiple levels including promotion and expansion of inhibitory immune cell subsets (Tregs and MDSC), inhibition of dendritic cell (DC) maturation, suppression of T cell responses, and immune cell trafficking across tumor endothelia [31–33]. Combination treatment of advanced melanoma patients with ipilimumab and bevacizumab led to intense infiltration of the tumor vasculature with CD8+ T cells and CD163+ dendritic macrophages, increased E-selectin expression (indicating endothelial activation), and higher numbers of circulating memory CD4+ and CD8+ T cells (CCR7^+/−^CD45RO^+^) in the peripheral blood [34]. The clinical benefit appeared promising compared to historical data with ipilimumab alone. Consequently, targeting angiogenesis may be an effective strategy to increase the efficacy of PD-1/PD-L1 inhibition. Several clinical trials investigating this combination are ongoing in multiple tumors types, including melanoma, renal cell cancer, and NSCLC [33].

#### Radiation therapy

Radiotherapy promotes the release or expression of tumor antigens in addition to immune adjuvant-like effects, leading to stimulation of immune responses. In animal models, this “in situ tumor vaccination”, while rarely effective as monotherapy, has shown synergy with various immunotherapy approaches including CTLA-4 blockade [35–37]. More recently, combined radiation and CTLA-4 blockade showed potential synergy in advanced melanoma patients [38]. Consistent with an effective vaccination, radiation when given with CTLA-4 blockade, induced diversification of the T cell receptor repertoire of TILs and shaped the repertoire of expanded T cell clones [39]. Resistance to radiation and CTLA-4 blockade was found to be mediated by upregulation of PD-L1, leading to T cell exhaustion. Taken together, the findings provide a rationale for combined PD-1 inhibition and radiotherapy.

#### Inhibition of IDO

IDO catalyzes the cleavage of L-tryptophan, resulting in the production of kynurenine. Depletion of tryptophan and accumulation of kynurenine metabolites led to increased numbers and function of Tregs and blocked the proliferation of effector T cells [40, 41]. IDO is expressed constitutively by tumor cells or by host immune cells such as macrophages and DCs in the tumor or lymph nodes [42, 43], but can also be induced by inflammatory cytokines such as IFN-γ during a tumor directed immune response, potentially mitigating the effectiveness of immunotherapy [44]. IDO has been implicated in promoting T cell resistance to anti-CTLA-4 Ab blockade in murine melanoma models. Combined inhibition of IDO and immune checkpoint blockade (CTLA-4, PD-1, and PD-L1) has shown T cell dependent synergy in melanoma and breast cancer mouse models. Based on this pre-clinical evidence, several IDO inhibitors are currently in clinical investigation as monotherapies and in combination with CTLA-4 and PD-1 inhibition. Promising response rates in NSCLC and melanoma with pembrolizumab plus the IDO inhibitor epacadostat were recently reported, leading to exploration of this combination in a phase III trial in melanoma (NCT02752074) and a recently announced expansion of this phase III program into NSCLC, renal, bladder, and head and neck cancers [45].

#### Chemotherapy

Chemotherapy-induced cancer cell death can promote tumor antigen presentation potentially leading to priming of tumor specific T cells in addition to its capacity to directly stimulate immune effectors and inhibit immune suppressive factors [46]. Therefore, chemotherapy has the potential to convert a non-inflamed tumor into an inflamed one and may thus lead to synergy with PD-1/PD-L1 inhibition particularly in non-inflamed, chemotherapy sensitive tumors.

#### Cytokines

Cytokines such as granulocyte macrophage colony-stimulating factor (GM-CSF) and interferon-α can promote DC function, leading to increased T cell priming and enhancing the activity of tumor directed T cells [47, 48]. The potential for additional clinical activity with CTLA-4 blockade has been shown with both GM-CSF and interferon-α in patients with advanced melanoma [49, 50]. Inhibition of immune suppressive cytokines such as TGF-β and IL-10 using neutralizing antibodies also has potential synergistic activity with PD-1/PD-L1 blockade. Other immune-potentiating cytokines that have shown activity against cancer include interleukin (IL)-2, IL-12, IL-15, IL-18, and IL-21. In a small phase I/II trial of high-dose IL-2 and escalating doses of ipilimumab in 36 patients with metastatic melanoma, a slightly higher response rate compared to historic monotherapy data (22%) was reported [51]. Of note, on further follow-up combination treatment was associated with an unanticipated 17% complete response rate. These data support further clinical studies that of combine cytokines and PD-1/PD-L1 blockade, and several such trials are currently underway.

## Pre-clinical murine models for testing of combination tumor immunotherapy

In contrast to cytotoxic and targeted therapy agents that directly kill tumor cells, tumor immunotherapy mediates tumor regression indirectly through activation of innate and adaptive host immune responses or by reversing tumor-mediated immune suppression. This implies that therapeutic responses may follow a more prolonged kinetic course and also may be associated with immune-related adverse events (irAEs), which are mediated by activated immune effector cells in various host tissues. Based on the unique mechanisms of tumor rejection and toxicity, murine models used for testing of tumor immunotherapy must incorporate interactions between established tumors, the host tumor microenvironment, and the immune system to fully evaluate the therapeutic and toxicity profiles of potential immunotherapy agents administered alone or in combination. Murine models are ideal for cancer research because tumors generally establish quickly, genetic manipulation of the host and tumors is relatively simple, and mice are easy to maintain, monitor, and assess. The characteristic features of the more commonly used murine tumor models are described in Table 1.

**Table 1:** Characteristic features of available pre-clinical murine tumor models

Model	Advantages	Disadvantages
Transplantable tumor • syngeneic murine • xenografts from human cancer cells lines • patient-derived xenografts (PDX)	• Tumors usually grow quickly • Reliable and reproducible • Can use different tumor cell lines • Gene expression easily manipulated in cell lines	• Rapid tumor growth may not allow time for physiologic immune system interactions • Does not mimic natural tumor formation • Tumor microenvironment is not relevant • Local injections can result in inflammation altering normal host response • Genetic engineering may create xenoantigens
Orthotopic tumor	• Allows normal tumor microenvironment to develop • Maintains many of the advantages of transplantable tumors	• Often grow quickly and do not allow interactions with immune system • May be challenging to get tumor injected or to establish in native tissue or organ
Spontaneous tumor • Carcinogen-induced • Genetically-mediated (GEMM)	• Tumors arise in situ • Tumors develop with host microenvironment • Tumors may have transgenic expression of oncogenes or inactivation of tumor suppressor genes • Tumors may exhibit more physiologic tumor growth kinetics and response to treatment • Assessment of toxicity is more relevant to humans	• Tumors may take more time to develop • Heterogeneity may arise and require more animals to determine therapeutic responses • Tumor induction may require carcinogens or genetic manipulations that alter the natural course of tumor development • Other cells may be affected • Tumor monitoring may be difficult
Immunodeficient mice	• Allows study of specific immune components • Can accept range of allogeneic and xenogeneic tumor cells • Can be used to introduce specific immune effector cells through adoptive transfer	• May be prone to infection and limited lifespan • May not be able to determine impact on intact immune system • Leakiness can result in unanticipated immune activity • May be sensitive to radiation and other treatments
Humanized mice	• Allow more rapid study of human tumors and human immune system • May more accurately replicate human tumor/immune system interactions	• Engraftment may be low • Murine immune system may interfere with human elements • Access to human samples can be challenging • Expensive

*GEMM* genetically-engineered mouse model*; PDX* patient-derived xenograft

The standard murine model utilizes a transplantable tumor system in which cultured cell lines derived from murine tumors of various origins can be injected, typically into the subcutaneous region of a mouse. These tumor cells must be derived in the same genetic background of the mouse and allows for rapid growth, simple growth assessment, and peripheral blood can be collected or mice can be euthanized at various times for toxicity analysis. These models are particularly useful for rapid studies of potential immunotherapy drug combinations and allow for inclusion of appropriate treatment controls. The model, however, has numerous limitations, including the lack of appropriate tumor microenvironment, potential problems with limited host immune system interactions since tumors often grow quickly, and local injection may induce inflammation that can influence tumor growth or drug response. Further, if the tumor cells harbor foreign transgenes, these may serve as xenoantigens and inadvertently promote tumor rejection. In some cases, murine tumor-associated antigens have been identified and these can be used to monitor immune responses and determine if tolerance can be broken during combination immunotherapy. While transplantable tumors are usually established in the subcutaneous location of the flanks, it is possible to inject the cells orthotopically, or in natural locations in which the tumor arises, to replicate the normal local environment. The potential importance of orthotopic models has been confirmed in at least one study in which the therapeutic efficacy of immunotherapy was less prominent when the cells were implanted into the kidney compared to subcutaneous tumors [52].

In order to better mimic human tumors, spontaneous tumor models have been developed in which tumors arise in the histologic tissue of origin, and these offer the benefit of more accurately reflecting patterns of tumor growth and treatment response kinetics. Such models may also be more appropriate for development and detection of irAEs. A major drawback to these models is that they generally require carcinogen induction or genetic manipulation, which may limit their clinical relevance. Examples of carcinogen-induced spontaneous tumors include methylcholanthrene (MCA)-induced fibrosarcomas and 7,12-dimethylbenz[a]anthracene (DMBA)/12-O-tetradecanoylphorbol-13-acetate (TPA)-induced skin papillomas [52, 53]. While these are more physiologically relevant, they often take longer for cancers to develop and may be associated with considerable heterogeneity requiring many more animals to obtain the required number of tumors and determine therapeutic activity. Further, establishing the timing of treatment may be particularly challenging since neoplastic transformation may occur over variable time periods with these models.

Genetically-engineered mouse models (GEMMs) utilize forced oncogene expression or knockout of known tumor suppressor genes, usually in a tissue-specific and/or temporally controlled manner [53, 54]. There are now several well-established, genetically mediated spontaneous tumor models in use. These include several breast cancer models in which selected oncogenic transgenes are driven by promoters that drive transgene expression in the mouse mammary epithelium [55]. The transgenes include Her-2/neu (ErbB2), polyoma middle T antigen (PyMT), simian virus 40 (SV40) T antigen, Ha-Ras, Wnt-1, TGF-α, and c-Myc. Oncogene expression in the MMTV-Neu and MMTV-PyMT mice is driven by the mouse mammary tumor virus promoter, and these mice develop multifocal mammary tumors and can exhibit metastatic disease in the lungs and lymph nodes, which typically occur after the first pregnancy. In contrast, the SV40 transgenic mice develop invasive tumors without the need for hormonal manipulation. In some cases, tumors require two genetic defects to promote tumor development.

There have been over 60 spontaneous murine models of melanoma reported to date [56, 57]. This includes a model in which the RET oncogene is fused to the metallothionein-I (MT) promoter-enhancer in a mixed murine strain background (C57BL/6xBALB/c), which produced systemic skin melanosis and spontaneous benign melanocytic tumors [58]. By backcrossing the mice over 10 times into C57BL/6 mice, a line in which melanocytic lesions progressed to invasive melanoma after several months was developed, and tumors eventually metastasize to lymph nodes and visceral organs. Bosenberg has developed an especially useful model in which melanoma is driven by both BRAF mutation and PTEN loss. These mice, characterized as Braf^CA^Tyr-creER^T2^Pten^fl/fl^, develop melanoma after exposure to 4-hydroxytamoxifen (4-HT), which induces de novo melanoma initiation [56]. A murine model of autochthonous lung tumors has been reported using adenoviral vectors encoding Cre recombinase, KRAS, and p53 in the pulmonary epithelia [59]. In general, the carcinogen-induced models are considered highly immunogenic with emergence of numerous neoantigens that can be recognized by the immune system. In contrast, models driven by germline mutations are typically not very immunogenic [60, 61].

Although it may seem counterintuitive, a number of immunodeficient murine models have been used to conduct mechanistic studies of immunotherapy. Today, there are numerous such models with selective as well as more global deficiencies in immune cells or immunologic function. These models can, thus, be classified as those with severe combined immunodeficiencies (SCID) and those with selective immunodeficiencies. The simplest immune deficient mouse was termed the nude (*nu*) mouse, in which thymic development is thwarted and results in deficient T cell maturation [62]. Nude mice were originally derived from mice with defects in the Forkhead box protein N1 (FOXN1) gene [63]. These mice typically live 6–12 months, accept xenografts and allow reasonable time for tumor treatment experiments. Nude females may be unable to nurse their young due to defects in mammary gland milk production and have largely been supplanted by SCID models.

A commonly used SCID model is based on knocking out the V(D)J recombination activation gene (RAG-1). RAG-1^−/−^ mice lack mature B and T cells, and these mice generally will not reject transplanted tumors [64]. The potential with these mice is that subpopulations of lymphocytes can be adoptively transferred, and the therapeutic impact of various treatment regimens can be assessed with and without specific lymphocyte populations. Because of this capability, these models have provided valuable insights into the mechanisms underlying antitumor immunity [65–67]. Another SCID model uses mice with mutations in the Prkdc gene, which encodes a protein that resolves DNA strand breaks during V(D)J recombination and results in the absence of functional B and T cells [68, 69]. These Prkdc^scid^ (also known as non-obese diabetic or NOD^scid^) mice do have a normal hematopoietic microenvironment, can accept both allogeneic and xenogeneic grafts, allow adoptive transfer experiments, and rarely develop mature lymphocytes. A particularly immunodeficient SCID model is the NSG mouse (NOD^scidgamma^; NOD.Cg-*Prkdc* ^*scid*^ *Il2rg* ^*tm1Wjl*^/SzJ), a strain of inbred mice in which the Prkdc gene and IL-2 receptor gamma gene, which is critical for IL-2-mediated signaling, are knocked out [70]. NSG mice lack both innate and adaptive immunity with loss of B cell, T cell, and NK cell function as well as reducedmacrophage and antigen-presenting cell function [71]. These mice are highly permissive for xenogeneic tumor engraftment and have been instrumental in studies of tumor immunotherapy and other human diseases. A variety of murine strains in which single molecular pathways are disrupted have also been generated and can be used for selective mechanistic studies.

The selection of immunodeficient murine strains for experimental studies depends on several features. The strain background is important as this may influence the H2 haplotype, tumor cell engraftment potential, and disease susceptibility. The NOD mice, for example, are prone to diabetes and lack innate immunity. The functional consequences of the genetic defects also need to be considered in strain selection. Some mice exhibit “leakiness”, in which the mice may start to generate functional immune cells as they age. The emergence of mature B and T cells has been reported in Prkdc^scid^ mice when they are older, especially if they are housed in non-specific pathogen-free conditions. Leakiness may also be more common in certain genetic backgrounds, such as C57BL/6 J and BALB/cByJ mice. The lifespan of individual mouse strains is also an important consideration as some immunodeficient mice die at a young age, with some becoming susceptible to thymic lymphomas, and this may limit their potential for long-term experiments. Some strains may also have difficulty breeding, as occurs with female nude mice, and this can limit usefulness. Some strains are highly sensitive to radiation (e.g., Prkdc^scid^ mice) and this can limit irradiation prior to engraftment or prevent studies of combination approached that utilize radiation therapy. Some mice also require pathogen-free environments, and husbandry capabilities may limit the choice of model. Finally, the impact of genetic mutations and how these influence cell function should be considered. For example, mutations in perforin can decrease NK cell activity whereas defects in the IL-2 receptor gamma chain can completely eliminate NK cell function.

The improved engraftment of human tumor cells in some of the SCID mouse models has allowed the generation of so-called humanized mouse models. These models further utilize the transfer of hematopoietic stem cells or, more recently autologous peripheral blood, to reconstitute the normal human immune system [72]. While several models have been proposed with considerable progress in replicating human immune-tumor components, there is still debate about how closely the humanized mice mimic the human host. Investigators are exploring the number of cells transferred, the route of transfer, the timing and age of transfer, and irradiation sources to optimize immune engraftment. Whether these mice truly organize a relevant tumor microenvironment remains unclear, but there is some evidence that mild graft-versus-host disease (GVHD) can develop, suggesting these models may be helpful for evaluating immune system activation and emergence of irAEs [72]. Other strategies in development include engineering expression of various cytokines into the mice to allow for more efficient immune function. Further refinements may be necessary before these mice can be endorsed as a significant improvement over other models. The generation of humanized mice is also complicated by the need for access to human tumor tissue and hematopoietic cells, institutional review board (IRB) approval, and often a need for rapid execution of cell transfer and frequently high cost to conduct experiments.

Many of the murine models have been helpful for evaluating therapeutic activity of monotherapy and combination immunotherapy agents, but few have faithfully replicated the toxicity profiles observed in humans [73–76]. It is possible, however, that the manifestations of irAEs may be present but challenging to detect in the murine models and may depend on the length of tumor establishment, background strain of the mouse used, or subtle impact of genetic changes in some models. Despite these limitations, there has been some progress in which autoimmune side effects have been observed. This includes the appearance of vitiligo in C57BL/6 mice bearing melanoma tumors and treated with a variety of immunotherapy strategies or the induction of hypophysitis in SJL/J mice treated with multiple treatments of CTLA-4 blockade [73, 74]. A strategy to better evaluate toxicity may be to use murine models in which the particular mouse strain is more susceptible to development of autoimmune symptoms (e.g., NOD, SJL/J, etc.). Another approach may be to add additional immune regulation to the model. For example, eradication of CD4 + FoxP3+ Tregs has been reported in the DEREG mouse model where mice have been engineered to express a diphtheria toxin (DT) receptor-enhanced green fluorescent protein fusion protein driven by the FoxP3 gene locus [75]. These mice permit conditional depletion of Tregs using injections of diphtheria toxin and some experiments have shown that DEREG mice may be more susceptible to autoimmune-related side effects with immunotherapy treatment [76].

The availability of numerous murine models that allow establishment of human tumors and immune system components provides an important resource for more rapidly testing rational combinations of immunotherapy agents. The large number of models further promotes more relevant systems to assess both therapeutic response and propensity for irAEs. While all models have limitations (see Table 1), the range of models allows selection of systems that most closely resemble the particular cancer, immunologic targets, and genetic factors that most closely mirror the human host and permit more rapid development of novel combination treatment strategies for clinical trials.

There has been considerable controversy regarding limitations, both real and perceived, in the utility of preclinical tumor models as tools to inform the clinical development of new oncology agents. While some limitations are certainly clear, in other instances, concerns may be driven by mouse models being used inappropriately or unrealistically, as opposed to intrinsic flaws in the models themselves. For several reasons, preclinical models may be particularly useful for cancer immunotherapy and in the development of new combination immunotherapy regimens [77]. Optimizing the dose, schedule, and configuration of immunotherapeutic combinations may be complex, yet as discussed above, is critical to additively or synergistically engage immunoregulatory mechanisms and maximize the risk-benefit profile of a given regimen. This may necessitate the comparison of a range of distinct schedules and configurations for combinations to maximize both their pharmacodynamic activity and their antitumor efficacy with acceptable tolerability. While the need for additional clinical optimization of dose and schedule is often inevitable, the assessment of new regimens in rigorous preclinical models may help to substantially focus the scope and cost of these efforts, and also may enable the interrogation of candidate clinical biomarkers to monitor the biologic activity of these combinations. Preclinical models may also enable more thorough understanding of the interaction between tumor and the host immune system in vivo, and may be utilized to enable rational, hypothesis-driven identification of mechanism-based combinations for clinical testing. In tandem with more rigorous early clinical development of combination regimens, preclinical models may play an important role in identifying and optimizing the safety, clinical activity, and overall risk-benefit profile of immunotherapeutic combinations.

## Safety and toxicology

### Combination therapy: proof-of-concept and lessons learned in patient safety

The pronounced clinical activity of checkpoint inhibitors including antibodies directed against CTLA-4 [2], PD-1 [4, 78] and PD-L1 [5, 79] has transformed the care of several cancers including melanoma, renal cell carcinoma, NSCLC, bladder cancer, head and neck cancer, Hodgkin lymphoma, and others. In turn, numerous preclinical studies have now demonstrated the synergistic potential of immunotherapeutic combinations [80–89]. However, it has also shown that substantive incremental toxicity can result from immunotherapeutic combinations, depending on both the patient population and the dose and schedule that is utilized [90–93]. In initial studies in patients with metastatic melanoma, marked enhancement of clinical activity was observed in patients treated with the combination of ipilimumab and nivolumab [90, 94] as reflected by the objective response rate (ORR), the kinetics and depth of tumor regression, and landmark rates of OS compared to historical experience with either ipilimumab or nivolumab alone. In subsequent randomized trials, the combination of ipilimumab and nivolumab has demonstrated superior progression-free survival (PFS) compared to ipilimumab alone in patients with melanoma [27, 28], and this combination has now been approved by the FDA for treatment-naive patients with melanoma. The initial phase I study of ipilimumab/nivolumab demonstrated grade 3/4 drug-related adverse events (AEs) in 53% of patients across the range of doses tested, while rates of grade 3/4 AEs in the subsequent randomized phase III were 55% in patients treated with the combination versus 27.3% or 16.3% among patients treated with either ipilimumab or nivolumab alone, respectively [27, 90]. Notably, although standard doses of ipilimumab (3 mg/kg) could be combined safely with doses of nivolumab up to 1 mg/kg, and standard doses of nivolumab (3 mg/kg) could be combined safely with doses of ipilimumab up to 1 mg/kg, combined administration of standard doses of both ipilimumab (3 mg/kg) and nivolumab (3 mg/kg) was poorly tolerated and exceeded the maximum tolerated dose (MTD) for the combination [90]. Nonetheless, despite the increase in the incidence of grade 3/4 AEs in patients treated with the combination of ipilimumab and nivolumab compared to either single agent alone, it is important to note that observed events were generally qualitatively similar for patients treated with combination therapy and the individual single agents. Further, the institution of algorithm-based supportive care has also been very effective in management of patients treated with checkpoint inhibitors and no treatment-related deaths were attributed to the combination of ipilimumab/nivolumab in the phase III study [27]. Collectively, these observations highlight the importance of flexible approaches to optimization of the dose and schedule of immunotherapeutic combinations. This requires rigorous clinical testing of various schedules for immunotherapeutic combination early in clinical development, and may require acceptance of the use of non-standard doses or schedules of individual agents to maximize the overall risk-benefit profile of a given combination. The importance of this consideration was further highlighted by a phase I study combining ipilimumab and the Raf inhibitor, vemurafenib, in patients with melanoma [95]. In this study, the initial cohort of patients was treated with standard doses of both ipilimumab (3 mg/kg) and vemurafenib (960 mg orally twice daily) administered concurrently, with plans for dose de-escalation in the event of dose-limiting toxicity (DLT) on this initial dose level. Substantial increases in toxicity, in particular hepatotoxicity, were observed in patients treated at this dose/schedule. Hepatotoxicity was also observed despite a reduction in the dose of vemurafenib (720 mg orally twice daily) in combination with the standard 3 mg/kg dose of ipilimumab. As a result, this trial was terminated very early, and there has been limited subsequent development of this combination. A recent follow-on study evaluated the combination of vemurafenib and ipilimumab using a sequential schedule of administration [96]. This regimen demonstrated a substantially improved safety profile, with marked reduction in hepatotoxicity compared to the prior study that administered ipilimumab and vemurafenib concurrently. These studies clearly highlight the clinical development challenges and risks in combining immuno-oncology agents at standard doses and schedules. Attempts to combine standard doses of these two highly active agents in patients with melanoma resulted in substantial incremental toxicity without improvements in clinical benefit, and further support the notion that when immunotherapy agents are used in combination or with conventional antineoplastic agents, it is reasonable to anticipate that compromises from standard dosing and schedules are likely to be required to unlock the therapeutic potential of combination regimens with acceptable risk-benefit. The potential for additional safety concerns might suggest that dose escalation, run-in, or sequential schemas should be considered in early phase clinical development of combination regimens.

Further, active combination regimens may have very distinct safety profiles in different patient populations, as illustrated by the experience using ipilimumab + nivolumab in patients with metastatic NSCLC [91]. Although this combination demonstrated potent antitumor activity and acceptable tolerability in patients with melanoma treated with ipilimumab/nivolumab at doses as high as ipilimumab (1 mg/kg) plus nivolumab (3 mg/kg) or ipilimumab (3 mg/kg) plus nivolumab (1 mg/kg) [90], the tolerability of this combination appeared to be quite distinct in patients with NSCLC. These same dosing regimens for ipilimumab and nivolumab were poorly tolerated in initial studies in patients with NSCLC despite elimination of the use of ipilimumab beyond induction, with 22/46 (48%) experiencing grade 3/4 AEs, 16 patients with treatment discontinuation due to AEs, and 3 drug-related deaths [91]. In addition, the overall ORR of 22% in this study was arguably no better than the 18% ORR achieved in phase I testing of nivolumab alone [4]. Additionally, a pilot study in 20 patients with glioblastoma demonstrated similar themes [92]. Here, patients were randomly assigned to treatment with either nivolumab monotherapy (3 mg/kg) every 2 weeks or an induction regimen consisting of ipilimumab (3 mg/kg) plus nivolumab (1 mg/kg) every 3 weeks, followed by nivolumab (3 mg/kg) monotherapy every 2 weeks. Drug-related grade 3/4 AEs were observed in 8/10 (80%) patients treated with the combination of ipilimumab and nivolumab, while drug-related AEs were all grade 1 or 2 in patients treated with nivolumab alone. Treatment-related discontinuations occurred in 5/10 (50%) patients treated with the combination compared to none in patients treated with nivolumab alone. Landmark 6-month OS rates were essentially the same in the combination (80%, 8/10 patients) and the nivolumab monotherapy (70%, 7/10 patients) arms.

Subsequent studies of ipilimumab and nivolumab in patients with NSCLC have now explored alternative combination regimens with lower dose intensity, with demonstration of both improved safety and enhanced clinical activity [97]. In this study, four distinct regimens were tested in patients with NSCLC, including Arm A: ipilimumab (1 mg/kg) plus nivolumab (1 mg/kg) administered every three weeks, Arm B: ipilimumab (1 mg/kg) every 6 weeks plus nivolumab (1 mg/kg) every 2 weeks, Arm C: ipilimumab (1 mg/kg) every 12 weeks plus nivolumab (3 mg/kg) every 2 weeks, and Arm D: ipilimumab (1 mg/kg) every 6 weeks plus nivolumab (3 mg/kg) every 2 weeks. All four of the arms were clinically active, with highly favorable ORR achieved for patients treated on Arm C (39%) and Arm D (31%) compared to the historical experience with either nivolumab or ipilimumab alone. The ORR for patients treated on Arm A and Arm B were 13% and 25%, respectively. Notably, not only were these alternative regimens highly active, but they were far better tolerated than the ipilimumab/nivolumab regimens established in patients with melanoma. The rate of grade 3/4 drug-related AEs ranged from 28 to 35% across the arms, with treatment-related discontinuations in less than 10% of patients, and no treatment-related deaths.

This collective experience with the ipilimumab/nivolumab combination highlights both the opportunity for patients using properly designed combination immunotherapy regimens, as well as the clinical development risks in not approaching the development of these regimens with both considerable flexibility and a rigorous approach to optimization of dose, schedule, and configuration of the respective agents. These studies demonstrate that regimented use of standard doses and schedules of agents based on monotherapy experience may lead to prohibitive toxicity and erroneous conclusions regarding the therapeutic potential and overall risk-benefit profile of immunotherapeutic combinations. In contrast, flexible investigation of non-standard doses and schedules early in the clinical development of combinations, may enable definition of regimens with additive or synergistic clinical activity with far more favorable safety profiles than when the same combinations are administered using the approved monotherapy doses and schedules of the respective agents. In addition, it appears clear that the optimal dose and schedule for a given combination may differ across distinct indications given differences in disease biology and/or co-morbidities in distinct patient populations.

### Safety considerations in early clinical testing

Immunotherapeutic combinations may present unique challenges that must be considered with respect to the assessment and management of patient safety. In general, immunotherapy agents demonstrate unique safety profiles that may differ considerably from the majority of conventional oncology drugs. For example, treatment with checkpoint inhibitors, including monoclonal antibodies that target CTLA-4, PD-1, or PD-L1 have been associated with a variety of autoimmune-like inflammatory phenomena that appear to be driven by disruption of self-tolerance to various normal tissues including thyroid, pituitary, liver, lung, colon, eye, and skin among others [98, 99]. Increased awareness of these events, recognition of the necessity of early diagnosis and intervention with immune-suppression, as well as the development of algorithm-based guidelines for the management of these irAEs has played a key role in enabling broad use of these agents in multiple tumor types with an acceptable safety profile. Other immunotherapeutic approaches including CAR T cells and CD3-based bispecific agents have been associated with systemic cytokine release syndrome (CRS) including fever, constitutional symptoms, and in severe instances, hemodynamic compromise [24, 100, 101]. Substantive improvement in the management of CRS has been afforded by meticulous supportive care, with early and aggressive immunosuppression as indicated, including the use of neutralizing anti-TNF and/or anti-IL-6 anti-cytokine antibodies. CAR T cell administration has also been associated with distinct, focal neurologic toxicity of uncertain etiology [102]. The experience derived from the management of AEs in patients treated with checkpoint inhibitors, cytokines, and CAR T cells has provided considerable insight that will enable future development of immunotherapy combinations. Common themes that have emerged from this experience highlight the importance of meticulous monitoring, early recognition and intervention with appropriate immune suppression, close collaboration between pharmaceutical sponsors and investigators in optimizing approaches for supportive care, and where appropriate, the implementation of algorithm-based supportive care regimens.

## Clinical trial design considerations

Historically, clinical development of many oncology combinations has proceeded using traditional development paradigms, where individual molecules undergo rigorous clinical testing as monotherapy, and often, combination trials have been deferred until clear monotherapy proof-of-concept has been established. As such, single agent phase I and phase II trials with each agent were typically executed, and only then, would phase I/II trials be triggered to investigate specific combinations. In many instances, failure to demonstrate substantive monotherapy activity led to cessation of further clinical development. This approach has been well-established using small molecules, but may be less well-suited for some immunotherapy agents, where clinical development efforts can be terminated prematurely based on unrealistic expectations for monotherapy activity using conventional criteria. More recently, driven by increasing recognition of the potential of immunotherapeutic combinations, as well as the reality that some agents may yield only modest clinical activity as monotherapy yet be highly active in the context of a therapeutic combination, innovative trial designs have been deployed increasingly to test these agents [103, 104]. These include run-in trial designs, zig-zag designs with or without de-escalation, and bifurcated designs among others. Run-in trial designs offer the prospect, where appropriate, for staged, sequential combination of an investigational agent with another standard-of-care drug, within the same patient after an initial monotherapy run-in window. At the study level, run-in trial designs may offer the prospect for getting an initial characterization of monotherapy safety before each patient is exposed to the combination, and conceptually, may enable more rapid identification of the MTD for a regimen depending on the extent of dose escalation. This approach may be most appropriate when there is particularly well-substantiated data suggesting that the safety profile of a given combination is anticipated to be highly favorable, and the respective agents clearly have non-overlapping safety profiles. Such studies can be executed with or without the option for de-escalation, but are likely used most appropriately when deployed with the flexibility for dose de-escalation of either agent. So-called “zig-zag” escalation trials afford the flexibility to explore various dose combinations of the respective agents by alternating the increases in dosing of each agent during dose escalation. This design may be particularly useful when the key driver of the clinical activity and/or safety of a given combination is less clear or when there is an anticipation that a given combination may have a narrower therapeutic window. Bifurcated designs also have been used recently for the investigation of some immunotherapeutic combinations. These are particularly well-suited for the testing of combinations where it is anticipated that an investigational agent will have a modest safety profile and limited potential for monotherapy clinical activity, yet there is clear rationale that this agent may synergize when administered in combination with another drug. In this approach, monotherapy dose escalation is executed through several dose levels with the novel agent. Presuming acceptable safety, the trial may then “bifurcate” down two distinct paths for subsequent escalation. On one arm, continued monotherapy escalation of the novel agent is pursued as appropriate until the MTD, maximum biologically-effective dose (MBED), or maximum administered dose (MAD) is defined. In parallel, escalation of the combination is pursued on a second arm, typically by combining a dose of the novel agent that is 1–2 dose levels below the highest monotherapy dose that already has been shown to be safe, in combination with another agent. Escalation can then proceed using either a fixed dose of the second agent or can proceed using a “zig-zag” approach guided by features of the specific combination. This approach allows for more rapid triggering of combination testing in clinical development, but may not be appropriate for some combinations. Further, a bifurcated-design trial should typically be structured so that the dose of the novel agent being tested in the combination arm does not exceed the dose that has been deemed safe in the monotherapy arm of the study.

We are entering an exciting era for combination immunotherapy that offers the prospect to build upon the powerful proof-of-principle established by the clinical experience with combination checkpoint blockade. Several key considerations may play an important role in enabling future progress with this approach. These include: a) rigorous assessment of the optimal dose, sequence, and schedule of agents in both preclinical models and the clinical setting; b) flexible approaches to decision-making in the selection of dose and schedule, and application of this decision-making across multiple indications; c) recognition that some agents may have limited monotherapy clinical activity, yet have high potential for clinical activity in the setting of an immunotherapy combination; d) careful attention to supportive care, including education of both patients and all members of the health-care team regarding the importance of early recognition and intervention for the management of irAEs; and e) the use of properly designed trials that enable efficient testing of the safety and clinical activity of combination immunotherapy regimens.

## Endpoints

### Safety

Novel combinations present the possibility of enhanced efficacy compared with monotherapies, yet also the real risk of additional or even novel toxicities. The above sections addressed the issues to consider in terms of trial design to incorporate appropriate observation periods and dosing levels needed to accommodate these issues. When considering combination therapies, management algorithms for each agent to be combined should be readily available. The lessons learned from the clinical development of ipilimumab + nivolumab have included a demonstration of no new toxicities with the combination compared with monotherapies, while more patients experienced multiple irAEs. The prior development of mechanistic management algorithms for both combinatorial partners allowed for the investigation of this combination in a global phase III trial with no treatment-related deaths in the combination group [27]. The investigations of ipilimumab with vemurafenib and ipilimumab with dacarbazine were also important sources of lessons regarding safety profile expectations. In both instances, hepatic enzyme elevation was more common than expected. Both of the non-immunologic partners were previously known to have low hepatic AE rates. Yet, when combined with the CTLA-4 blocking antibody, this toxicity was considerably more common [3, 95]. This should be kept in mind when considering cross-modality combinations.

### Efficacy

Early in the development of ipilimumab, it became apparent that conventional radiographic response criteria were not capturing the full spectrum of biologic activity of the agent. Some patients were demonstrating atypical response with clear disease progression before significant response occurred and mixed responses with regression of index lesions despite the appearance of new areas of disease. Using modified World Health Organization (mWHO) or Response Evaluation Criteria in Solid Tumors (RECIST) assessment, either pattern is considered progressive disease. The underlying mechanism could be either transient enlargement due to lymphocytic infiltration or truly a delay in response due to the need for multiple epitopes to be recognized and responded to. With careful analysis, it was estimated that 15–25% of melanoma patients treated with ipilimumab who initially were classified as having disease progression, eventually had response or long-term disease stabilization and demonstrated long-term survival. This pattern has also been reported with the oncolytic virus, T-VEC, in patients with melanoma [20]. These observations led to the development of a proposed set of new response criteria, the immune-related response criteria (irRC) [105, 106]. While the irRC are still considered non-validated and exploratory, it is also clear that a subset of patients treated with other immunotherapies (PD-1 pathway blocking agents) manifest similarly atypical response kinetics, albeit less frequently [107]. Given the clinical imperative for rapid drug development, the use of OS is becoming less practical as a sole primary endpoint. Therefore, combination studies have employed PFS and OS as co-primary endpoints as a means to capture early signals of high activity while also maintaining the importance of OS as the true measure of durability that is expected from immunotherapy. In the phase III study of T-VEC a primary clinical endpoint of durable response rate (DRR) was used, which incorporated both response rate (based on mWHO criteria) and time (duration ≥ 6 months).

### Registration pathway

The registration pathway for combination therapies can be considered in a variety of ways. The ipilimumab registration study (MDX-010-20) followed a ‘contribution of components’ model for testing ipilimumab, gp100 peptides or the combination. The study hypothesized that the combination would be superior to either monotherapy. In the end, the two ipilimumab-containing groups had similar OS. The presence of all three groups allowed for the ipilimumab monotherapy group to be evaluated for OS vs gp100 alone, therefore allowing for the possibility of demonstrating activity for multiple groups. This type of trial design is a clean route to prospectively assess combinations in the context of monotherapies but does lead to large studies. Other considerations in registration pathway designs include weighing the value of concurrent versus sequential schedules. Another niche for combination drug development is the ‘add-in’ design where a new agent is added at the time of progression on the ‘foundational’ agent. Given that all patients treated with the combination will have progressive disease when beginning combination therapy, a modest degree of clinical activity with the additional agent could represent a rapid route to registration. One other consideration in designing combination registration pathways is that combination immunotherapy may have a different degree of tolerability in patients with different malignancies. Therefore, flexibility in dose levels and frequency needs to be considered when evaluating combinations across different disease types. Early and more frequent dialogue with regulatory agencies may also be helpful in designing and conducting combination immunotherapy clinical trials.

## Conclusions

The number of cancer patients who benefit from immunotherapy has increased due to a better understanding of the immune response to cancer along with recent advances in biomarker development. The goal of combination approaches is to expand the spectrum of patients who respond to cancer immunotherapy (more responding patients in tumors that are sensitive to monotherapy and the identification of new sensitive tumor types that do not respond to monotherapy alone) and to improve the quality of clinical responses (i.e., extension of response duration, PFS, and OS) beyond what can be achieved with monotherapy alone. With research to further elucidate the mechanisms of action behind these agents as well as increased understanding of the resistant counter defense employed by tumors, the development of rational combination approaches is now extending even beyond doublets. Novel triplet regimens of synergistic combinations of immunotherapy agents as well as immunotherapy with conventional or targeted therapies are being investigated in a variety of disease settings. There is tremendous potential for these approaches to extend the clinical success of immunotherapies. However, the added benefit of each additional drug must be properly evaluated against the added toxicities as well as economic impact of the cost of these strategies (the “value proposition”). Previous experience also demonstrates that combination approaches should not be developed solely on the standard dosing and regimens of single agents. Instead, there is a need to investigate the dose and schedule of combination immunotherapies thoroughly and with added flexibility to optimize the dose, schedule, and configuration of each agent. Moreover, the optimal dose and schedule for a given combination may differ across disease indications. Although pre-clinical animal models have limitations, they can be useful for assessing the therapeutic potential of specific combination regimens, interrogating the mechanism of action, and providing insight into the underlying biology of various therapeutic strategies. Progress in combination immunotherapy will also depend on thorough clinical testing, with proper clinical trial design and endpoints. The SITC Combination Therapies Taskforce has summarized the critical issues currently facing the clinical development of combination immunotherapy (Table 2). These issues should help focus further development and promote discussion among academic, industry, and regulatory partners to more fully realize the potential of combination immunotherapy for the treatment of cancer.

**Table 2:** Critical issues in combination immunotherapy drug development

• Combination regimens should be based on scientific evidence of underlying tumor cell and immune system biology whenever possible. There is evidence that combinations within drug classes (e.g., T cell checkpoint inhibitors) and across classes may be clinically beneficial. • Murine models have limitations but may be useful for early proof-of-principle for specific combination strategies and can be useful for understanding the biology and mechanisms of action for certain combinations. These models have generally not been useful for toxicity assessment. • While combination therapy may be anticipated to improved therapeutic effectiveness, the approach may also increase the potential for adverse events. This possibility should be carefully considered in developing clinical study designs for combination immunotherapy. • Innovative clinical study designs may be useful for early phase combination immunotherapy development. These may include dose escalation, dose de-escalation, zig-zag, run-in and sequential administration designs. A better understanding of the adverse event profile, as may be obtained from small monotherapy phase I studies, is helpful in optimizing the design of combination trials. • The unique mechanism of action for immunotherapy agents suggests that new clinical endpoints may be needed for early phase drug development. The use of progressionfree survival may be misleading given the delayed kinetics of response that can occur with some agents and regimens. New endpoints, such as immune-related response criteria or durable response rate, may be better for predicting clinical benefit in late stage studies. • Early and frequent discussion with regulatory agencies should be considered for combination immunotherapy regimens. • Other issues, such as intellectual property, conflict of interest, quality of life, patientreported outcomes, and the financial costs vs. overall health benefits as defined in the value proposition of combination immunotherapy, will be important issues for further discussion.	

## Abbreviations

4-HT: 4-hydroxytamoxifen
AE: Adverse events
CAR: Chimeric-antigen receptor
CRS: Cytokine release syndrome
CTLA-4: Cytotoxic T lymphocyte associated protein 4
DC: Dendritic cell
DLT: Dose-limiting toxicity
DMBA: 7,12-dimethylbenz[a]anthracene
DRR: Durable response rate
DT: Diphtheria toxin
FOXN1: Forkhead box protein N1
GEMM: Genetically engineered mouse model
GITR: Glucocorticoid-induced tumor necrosis factor receptor-related protein
GVHD: Graft-versus-host disease
IDO: Indoleamine 2,3-dioxgenase
IL: Interleukin
irAE: Immune-related adverse event
IRB: Institutional review board
irRC: Immune-related response criteria
MAD: Maximum administered dose
MBED: Maximum biologically-effective dose
MCA: Methylcholanthrene
MDSC: Myeloid derived suppressor cells
MSI: Microsatellite instability
MT: Metallothionein-I
MTD: Maximum tolerated dose
mWHO: Modified World Health Organization
NK: Natural killer cell
NKT: Natural killer T cell
NSCLC: Non-small cell lung cancer
ORR: Objective response rate
OS: Overall survival
PD-1: Programed cell death 1
PD-L1: Programed cell death ligand 1
PFS: Progression-free survival
PyMT: Polyoma middle T antigen
RAG-1: Recombination activation gene
RECIST: Response evaluation criteria in solid tumors
SCID: Severe combined immunodeficiencies
SITC: Society for Immunotherapy of Cancer
SV40: Simian virus 40
TPA: 12-O-tetradecanoylphorbol-13-acetate
Treg: Regulatory T cell
T-VEC: Talimogene laherparepvec
